# Association between *AIRE* gene polymorphism and rheumatoid arthritis: a systematic review and meta-analysis of case-control studies

**DOI:** 10.1038/s41598-017-14375-z

**Published:** 2017-10-26

**Authors:** Bálint Bérczi, Gellért Gerencsér, Nelli Farkas, Péter Hegyi, Gábor Veres, Judit Bajor, László Czopf, Hussain Alizadeh, Zoltán Rakonczay, Éva Vigh, Bálint Erőss, Kata Szemes, Zoltán Gyöngyi

**Affiliations:** 1 Department of Public Health Medicine, Medical School, University of Pécs, Pécs, Hungary; 2 Institute of Bioanalysis, Medical School, University of Pécs, Pécs, Hungary; 3 MTA-SZTE Translational Gastroenterology Research Group, Szeged, Hungary; 4 Institute for Translational Medicine, University of Pécs, Pécs, Hungary; 5 Department of Translational Medicine, First Department of Medicine, University of Pécs, Pécs, Hungary; 6 1st Department of Pediatrics, Semmelweis University, Budapest, Hungary; 7 Department of Gastroenterology, First Department of Medicine, University of Pécs, Pécs, Hungary; 8 Division of Cardiology and Angiology, First Department of Medicine, University of Pécs, Pécs, Hungary; 9 Department of Haematology, First Department of Medicine, University of Pécs, Pécs, Hungary; 10 Department of Pathophysiology, University of Szeged, Szeged, Hungary; 11 Department of Radiology, Medical School, University of Pécs, Pécs, Hungary

## Abstract

Autoimmune regulator (AIRE) is a transcription factor that functions as a novel player in immunological investigations. In the thymus, it has a pivotal role in the negative selection of naive T-cells during central tolerance. Experimental studies have shown that single nucleotide polymorphism (SNP) alters transcription of the *AIRE* gene. SNPs thereby provide a less efficient negative selection, propagate higher survival of autoimmune T-cells, and elevate susceptibility to autoimmune diseases. To date, only rheumatoid arthritis (RA) has been analysed by epidemiological investigations in relation to SNPs in *AIRE*. In our meta-analysis, we sought to encompass case-control studies and confirm that the association between SNP occurrence and RA. After robust searches of Embase, PubMed, Cochrane Library, and Web of Science databases, we found 19 articles that included five independent studies. Out of 11 polymorphisms, two (rs2075876, rs760426) were common in the five case-control studies. Thus, we performed a meta-analysis for rs2075876 (7145 cases and 8579 controls) and rs760426 (6696 cases and 8164 controls). Our results prove that rs2075876 and rs760426 are significantly associated with an increased risk of RA in allelic, dominant, recessive, codominant heterozygous, and codominant homozygous genetic models. These findings are primarily based on data from Asian populations.

## Introduction

Rheumatoid arthritis (RA) is a common autoimmune disease associated with chronic synovial inflammation. The resultant symmetrical polyarticular arthritis, combined with extra-articular complications, leads to functional impairment. In developed countries, disease prevalence is 0.5–1% of the adult population, and the annual incidence has been reported to be 5–50 per 100,000^1^. Although the aetiology of RA has not been completely elucidated, numerous publications agree that autoimmune T-cells may escape from the adaptive immune system and, by migrating to the synovium, initiate disease development^2–5^. RA susceptibility is determined by multiple environmental and genetic factors, including several risk alleles. The latest trans-ethnic genome wide association study (GWAS), which involved 29,980 RA cases and 73,578 controls, completely screened novel polymorphisms in genes contributing to the disease^6^. One of the associated genes that seems to play a pivotal role in controlling autoimmunity is autoimmune regulator (*AIRE*). The gene is located in the 21q22.3 region, is ∼12.5 kb long, and encodes a 545 amino acid protein of 58 kDa by 14 exonial sequences^7–9^. The AIRE protein is a transcription factor that is indispensable with regards to the negative selection of immature T-cells (thymocytes). Cooperating with DNA-binding proteins, AIRE controls the promiscuous expression of peripheral tissue antigens (PTA). Mutations in the protein coding gene sequence of *AIRE* results in the development of autoimmune polyendocrinopathy candidiasis-ectodermal dystrophy, an autoimmune deterioration of numerous organs^10–12^.

To date, increasing numbers of publications have suggested that SNPs in the gene sequence affect *AIRE* transcription. The SNPs thereby alter the functional activity of AIRE and potentially elevate disease susceptibility^7^. A recent experimental study described two distinct SNPs of *AIRE*. *AIRE*−230Y, and *AIRE*−655G. *AIRE*−230T haplotype transcriptionally modifies *AIRE* expression and influence negative selection, elevating the risk of autoimmunity^13^. Various SNPs in the *AIRE* genetic sequence have garnered attention; however, to date, only a minority of case-control studies have observed an association between gene polymorphism and susceptibility to diseases, including vitiligo^7,14^, alopecia areata^7,15^, melanoma^7,16^, systemic sclerosis^7,17^ and RA^7,18–22^. Among the latter diseases, only RA has been analysed by multiple case-control studies and, therefore, seems to be optimal to analyse positive or negative associations^7^. Xu *et al*. have published that *AIRE* polymorphism was associated with the increased risk of RA^23^. Here, we present a systematic review and first meta-analysis that includes case-control studies to verify the association of SNPs rs2075876 and rs760426 in the *AIRE* gene with RA.

## Results

### Characteristics of included studies

**Figure 1:**
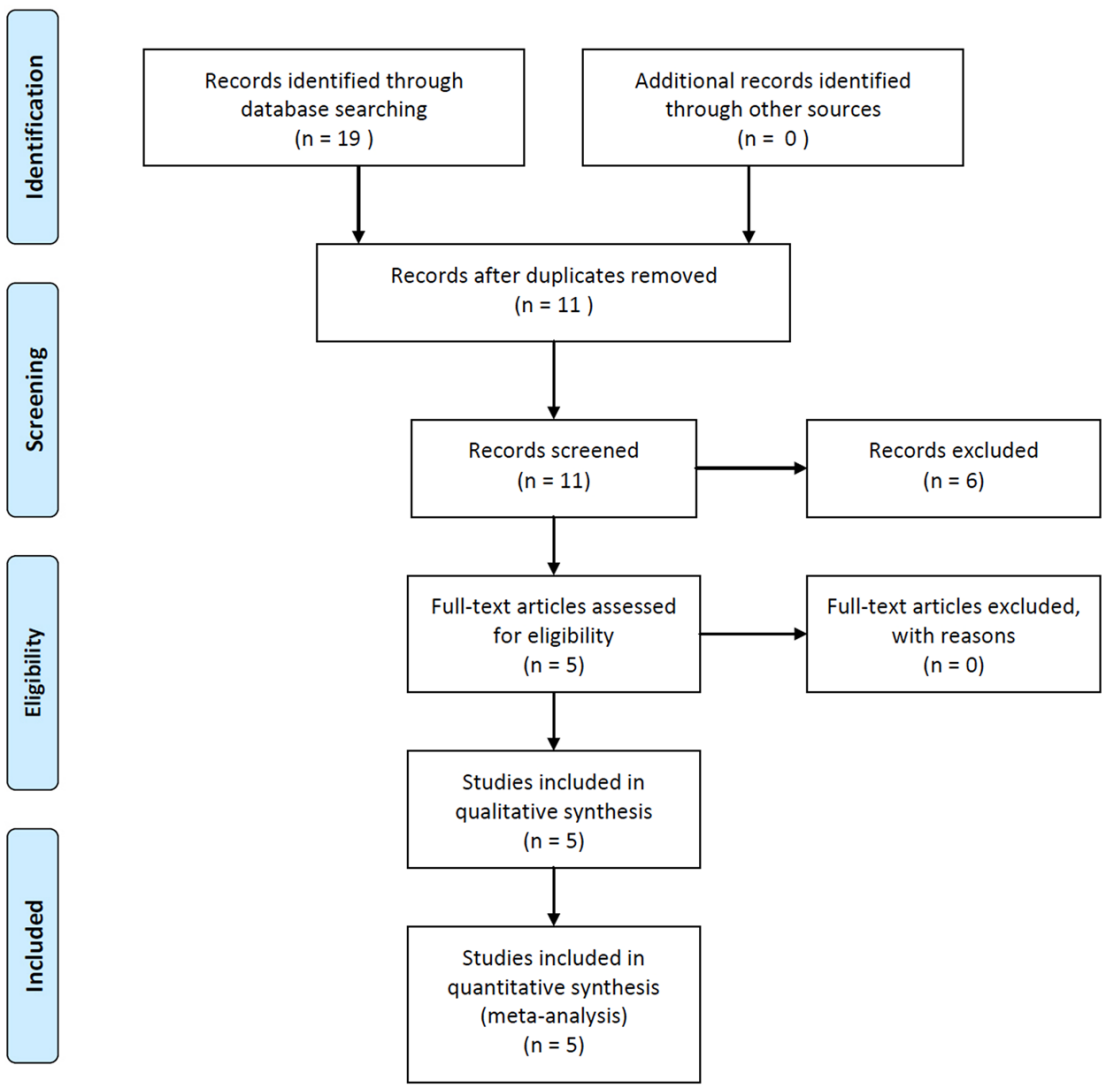
PRISMA flow diagram for inclusion and exclusion of studies in the meta-analysis.

We identified 19 publications after a thorough search of Embase, PubMed, Cochrane Library, and Web of Science databases. After removing duplicates, we reviewed the remaining 11 studies for eligibility and selected five publications for inclusion in our meta-analysis. Our PRISMA flow chart of the searching process is shown in Fig. 1. Asian and Caucasian ethnicities were involved. Diagnosis of RA was determined according to the American College of Rheumatology classification criteria in 1987^24^. The overall mean age of RA patients was 54.1 ± 2.4 years, and the percentage of female cases was 73.34%. Genotyping was conducted by microarrays, single base extension methods (SNaPshot), and Taqman SNP Genotyping Assays. By further reviewing the five eligible publications, we identified 11 SNPs of the *AIRE* gene (rs2075876, rs760426, rs1800250, rs2776377, rs878081, rs1055311, rs933150, rs1003854, rs2256817, rs374696, rs1078480). Only rs2075876 and rs760426 were involved in four or more studies; therefore, we performed meta-analysis for rs2075876 (7145 cases and 8579 controls) and rs760426 (6696 cases and 8164 controls). All genotype frequencies of the controls were in Hardy-Weinberg Equilibrium (HWE). Characteristics of the included studies on rs2075876 and rs760426 are summarized in Table 1.

**Table 1:** Characteristics of the included studies on SNP rs2075876 (G > A) and rs760426 (A > G) (SNP = single nucleotide polymorphism; NA = not available; HB = hospital based).

			Year	Country	Ethnicity	Diagnostic criteria	Genotyping	Mean age		Female %		Control source
								case	control	case	control	
*SNP rs2075876(G* > *A)*	Terao C	A	2011	Japan	Asian	American College of Rheumatology for RA (1987)	microarrays	63.0 ± 12.5	52.0 ± 15.8	82.1	60.6	HB
		B	2011				microarrays	60.8 ± 11.5	38.1 ± 11.9	84.1	39.6	HB
		C	2011				microarrays	61.4 ± 11.5	52.5 ± 15.2	81.4	44.4	HB
	García-Lozano JR		2013	Spain	Caucasian	American College of Rheumatology for RA (1987)	Taqman SNP genotyping assay	49.2 ± 14.8	NA	74.3	NA	HB
	Shao S		2014	China	Asian	American College of Rheumatology for RA (1987)	SNaPshot assay	48.7 ± 14.2	47.0 ± 16.3	80.6	37.0	HB
	Feng ZJ		2015	China (Han)	Asian	American College of Rheumatology for RA (1987)	Taqman SNP genotyping assay	54.1 ± 11.2	52.4 ± 11.8	53.5	58.5	HB
	Li X		2016	China (Shaanxi)	Asian	American College of Rheumatology for RA (1987)	Snapshot Assay	43.5 ± 19.2	44.3 ± 17.8	64.3	59.7	HB
*SNP rs760426 (A* > *G)*	Terao C	A	2011	Japan	Asian	American College of Rheumatology for RA (1987)	microarrays	63.0 ± 12.5	52.0 ± 15.8	82.1	60.6	HB
		B	2011				microarrays	60.8 ± 11.5	38.1 ± 11.9	84.1	39.6	HB
		C	2011				microarrays	61.4 ± 11.5	52.5 ± 15.2	81.4	44.4	HB
	Shao S		2014	China	Asian	American College of Rheumatology for RA (1987)	SNaPshot assay	48.7 ± 14.2	47.0 ± 16.3	80.6	37.0	HB
	Feng ZJ		2015	China (Han)	Asian	American College of Rheumatology for RA (1987)	Taqman SNP genotyping assay	54.1 ± 11.2	52.4 ± 11.8	53.5	58.5	HB
	Li X		2016	China (Shaanxi)	Asian	American College of Rheumatology for RA (1987)	Snapshot Assay	43.5 ± 19.2	44.3 ± 17.8	64.3	59.7	HB

### Meta-analysis of SNP rs2075876 (G > A)

**Figure 2:**
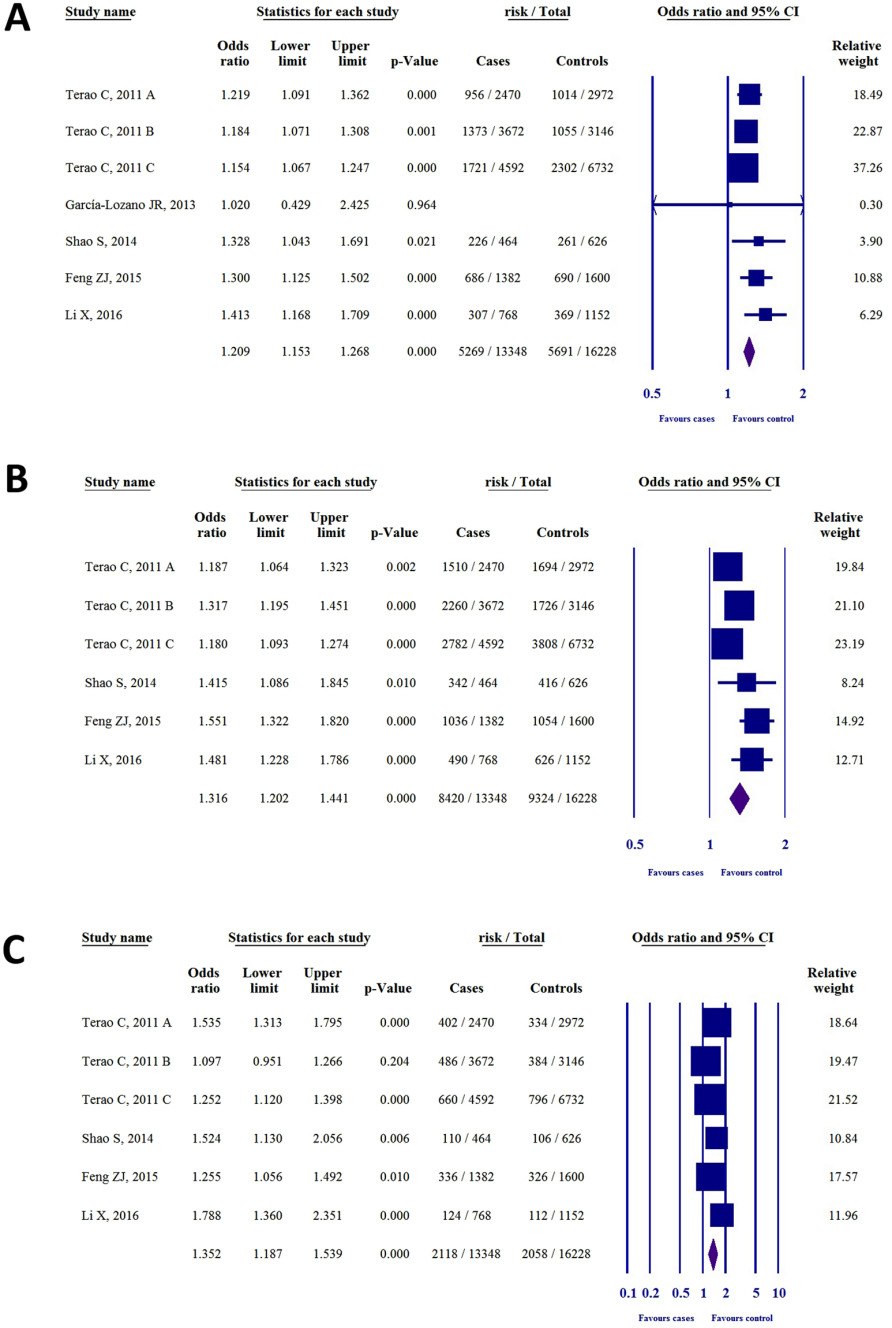
The association of SNP rs2075876 (G > A) with RA risk in different genetic models. (**A**) Allelic model (A vs. G). (**B**) Dominant model (AG + AA vs. GG). (**C**) Recessive model (AA vs. AG + GG).

Five studies were identified that investigated the association between SNP rs2075876 and RA susceptibility^18–22^. Most of the publications doubled the individual number to account for alleles; thus, to normalize the data, we also calculated with duplicated values (see Supplementary Table S1). GWAS by *Terao et al*.^18^ served as three independent case-control studies (denoted with A, B, C). With the exception of *García-Lozano et al*.^19^, all of the studies described the genotype distribution for GG, AG, AA. Therefore, we calculated odds ratios (ORs) for genetic models where there was no available or feasible data in the given study (Table 2). Results for each genetic model are shown in Fig. 2. For the allelic model (A vs. G, Fig. 2A) the ORs were 1.21 (95% CI 1.09–1.36, P < 0.001), 1.18 (1.07–1.30, P = 0.001), 1.15 (1.06–1.24, P < 0.001), 1.02 (0.42–2.42, P = 0.964), 1.32 (1.04–1.69, P = 0.021), 1.30 (1.12–1.50, P < 0.00), and 1.41 (1.16–1.70, P < 0.001). For the dominant model (AG + AA vs. GG, Fig. 2B) the ORs were 1.18, (95% CI 1.06–1.32, P = 0.002), 1.31 (1.19–1.45, P < 0.001), 1.18 (1.09–1.27, P < 0.001), 1.41 (1.08–1.84, P = 0.010), 1.55 (1.32–1.82, P < 0.001), and 1.48 (1.22–1.78, P < 0.001). For the recessive model (AA vs. AG + GG, Fig. 2C) the ORs were 1.53 (95% CI 1.31–1.79, P < 0.001), 1.09 (0.95–1.26, P = 0.204), 1.25 (1.12–1.39, P < 0.001), 1.52 (1.13–2.05, P = 0.006), 1.25 (1.05–1.49, P = 0.010), and 1.78 (1.36–2.35, P < 0.001). For the codominant heterozygous model (AG vs. GG, see Supplementary Fig. S1), the ORs were 1.08 (0.96–1.21, P = 0.168), 1.32 (1.20–1.47, P < 0.001), 1.13 (1.05–1.23, P = 0.002), 1.28 (0.97–1.70, P = 0.077), 1.51 (1.28–1.79, P < 0.001), and 1.34 (1.10–1.64, P = 0.003). For the codominant homozygous model (AA vs. GG, see Supplementary Fig. S1) the ORs were 1.60 (95% CI 1.35–1.89, P < 0.001), 1.27 (1.09–1.48, P = 0.002), 1.34 (1.19–1.51, P < 0.001), 1.78 (1.26–2.52, P = 0.001), 1.62 (1.32–1.99, P < 0.001), and 2.09, (1.56–2.81, P < 0.001).

**Table 2:** ORs, 95% CIs, and P-values for each genetic model in the association of SNPs rs2075876 (G > A) and rs760426 (A > G) with RA risk (NA = not available; OR = odds ratio; CI = confidence interval; *literature data.

polymorphism	study		Genetic model		OR	95%CI	P
***SNP rs2075876*** **(** ***G*** > ***A*** **)**	Terao C, 2011	A	Allelic*	(A vs. G)	1.21	1.09–1.36	<0.001
			Dominant	(AG + AA vs. GG)	1.18	1.06–1.32	0.002
			Recessive	(AA vs. AG + GG)	1.53	1.31–1.79	<0.001
			Codominant heterozygous	(AG vs. GG)	1.08	0.96–1.21	0.168
			Codominant homozygous	(AA vs. GG)	1.60	1.35–1.89	<0.001
		B	Allelic*	(A vs. G)	1.18	1.07–1.30	<0.001
			Dominant	(AG + AA vs. GG)	1.31	1.19–1.45	<0.001
			Recessive	(AA vs. AG + GG)	1.09	0.95–1.26	0.204
			Codominant heterozygous	(AG vs. GG)	1.32	1.20–1.47	<0.001
			Codominant homozygous	(AA vs. GG)	1.27	1.09–1.48	0.002
		C	Allelic*	(A vs. G)	1.15	1.06–1.24	<0.001
			Dominant	(AG + AA vs. GG)	1.18	1.09–1.27	<0.001
			Recessive	(AA vs. AG + GG)	1.25	1.12–1.39	<0.001
			Codominant heterozygous	(AG vs. GG)	1.13	1.05–1.23	0.002
			Codominant homozygous	(AA vs. GG)	1.34	1.19–1.51	<0.001
	García-Lozano JR, 2013		Allelic	(A vs. G)	1.02	0.42–2.42	0.964
			Dominant	(AG + AA vs. GG)	NA		
			Recessive	(AA vs. AG + GG)			
			Codominant heterozygous	(AG vs. GG)			
			Codominant homozygous	(AA vs. GG)			
	Shao S, 2014		Allelic*	(A vs. G)	1.32	1.04–1.69	0.021
			Dominant	(AG + AA vs. GG)	1.41	1.08–1.84	0.010
			Recessive	(AA vs. AG + GG)	1.52	1.13–2.05	0.006
			Codominant heterozygous	(AG vs. GG)	1.28	0.97–1.70	0.077
			Codominant homozygous	(AA vs. GG)	1.78	1.26–2.52	0.001
	Feng ZJ, 2015		Allelic	(A vs. G)	1.30	1.12–1.50	<0.001
			Dominant	(AG + AA vs. GG)	1.55	1.32–1.82	<0.001
			Recessive	(AA vs. AG + GG)	1.25	1.05–1.49	0.010
			Codominant heterozygous	(AG vs. GG)	1.51	1.28–1.79	<0.001
			Codominant homozygous	(AA vs. GG)	1.62	1.32–1.99	<0.001
	Li X, 2016		Allelic*	(A vs. G)	1.41	1.16–1.70	<0.001
			Dominant	(AG + AA vs. GG)	1.48	1.22–1.78	<0.001
			Recessive	(AA vs. AG + GG)	1.78	1.36–2.35	<0.001
			Codominant heterozygous	(AG vs. GG)	1.34	1.10–1.64	0.003
			Codominant homozygous	(AA vs. GG)	2.09	1.56–2.81	<0.001
***SNP rs760426*** **(** ***A*** > ***G*** **)**	Terao C, 2011	A	Allelic*	(G vs. A)	1.23	1.10–1.37	<0.001
			Dominant	(GG + GA vs. AA)	1.16	1.04–1.29	0.007
			Recessive*	(GG vs. GA + AA)	1.66	1.43–1.94	<0.001
			Codominant heterozygous*	(GA vs. AA)	1.03	0.92–1.16	0.582
			Codominant homozygous*	(GG vs. AA)	1.69	1.43–2.00	<0.001
		B	Allelic*	(G vs. A)	1.13	1.02–1.25	0.011
			Dominant	(GG + GA vs. AA)	1.19	1.08–1.31	<0.001
			Recessive	(GG vs. GA + AA)	1.15	1.00–1.32	0.047
			Codominant heterozygous	(GA vs. AA)	1.17	1.06–1.30	0.002
			Codominant homozygous	(GG vs. AA)	1.25	1.08–1.46	0.003
		C	Allelic*	(G vs. A)	1.16	1.08–1.26	<0.001
			Dominant	(GG + GA vs. AA)	1.18	1.09–1.27	<0.001
			Recessive	(GG vs. GA + AA)	1.19	1.07–1.32	0.001
			Codominant heterozygous	(GA vs. AA)	1.15	1.06–1.25	0.001
			Codominant homozygous	(GG vs. AA)	1.29	1.15–1.44	<0.001
	Shao S, 2014		Allelic*	(G vs. A)	1.25	0.98–1.60	0.062
			Dominant	(GG + GA vs. AA)	1.19	0.92–1.55	0.171
			Recessive	(GG vs. GA + AA)	1.55	1.16–2.08	0.003
			Codominant heterozygous	(GA vs. AA)	1.04	0.79–1.38	0.741
			Codominant homozygous	(GG vs. AA)	1.60	1.15–2.24	0.006
	Feng ZJ, 2015		Allelic*	(G vs. A)	1.87	1.09–2.45	0.074
			Dominant	(GG + GA vs. AA)	NA		
			Recessive	(GG vs. GA + AA)			
			Codominant heterozygous	(GA vs. AA)			
			Codominant homozygous	(GG vs. AA)			
	Li X, 2016		Allelic*	(G vs. A)	1.25	1.04–1.52	0.018
			Dominant	(GG + GA vs. AA)	1.32	1.10–1.59	0.003
			Recessive	(GG vs. GA + AA)	1.36	1.05–1.77	0.020
			Codominant heterozygous	(GA vs. AA)	1.26	1.03–1.54	0.020
			Codominant homozygous	(GG vs. AA)	1.54	1.16–2.04	0.003

Results of heterogeneity analysis for each genetic model are shown in Supplementary Table S2. For the allelic model P_h_ = 0.439 and I^2^ = 0%, for the dominant model P_h_ = 0.011 and I^2^ = 66.2%, for the recessive model P_h_ = 0.005 and I^2^ = 69.9%, for the codominant heterozygous model P_h_ = 0.004 and I^2^ = 70.5%, and for the codominant homozygous model P_h_ = 0.012 and I^2^ = 65.4%. Moderate heterogeneity was found in dominant, recessive, codominant heterozygous, and codominant homozygous models.

Only four out of 31 ORs were statistically insignificant, and the ORs revealed that SNP rs2075876 (G > A) is associated with an elevated risk of RA. These results therefore suggest a link between *AIRE* SNP rs2075876 (G > A) and RA susceptibility.

### Meta-analysis of SNP rs760426 (A > G)

**Figure 3:**
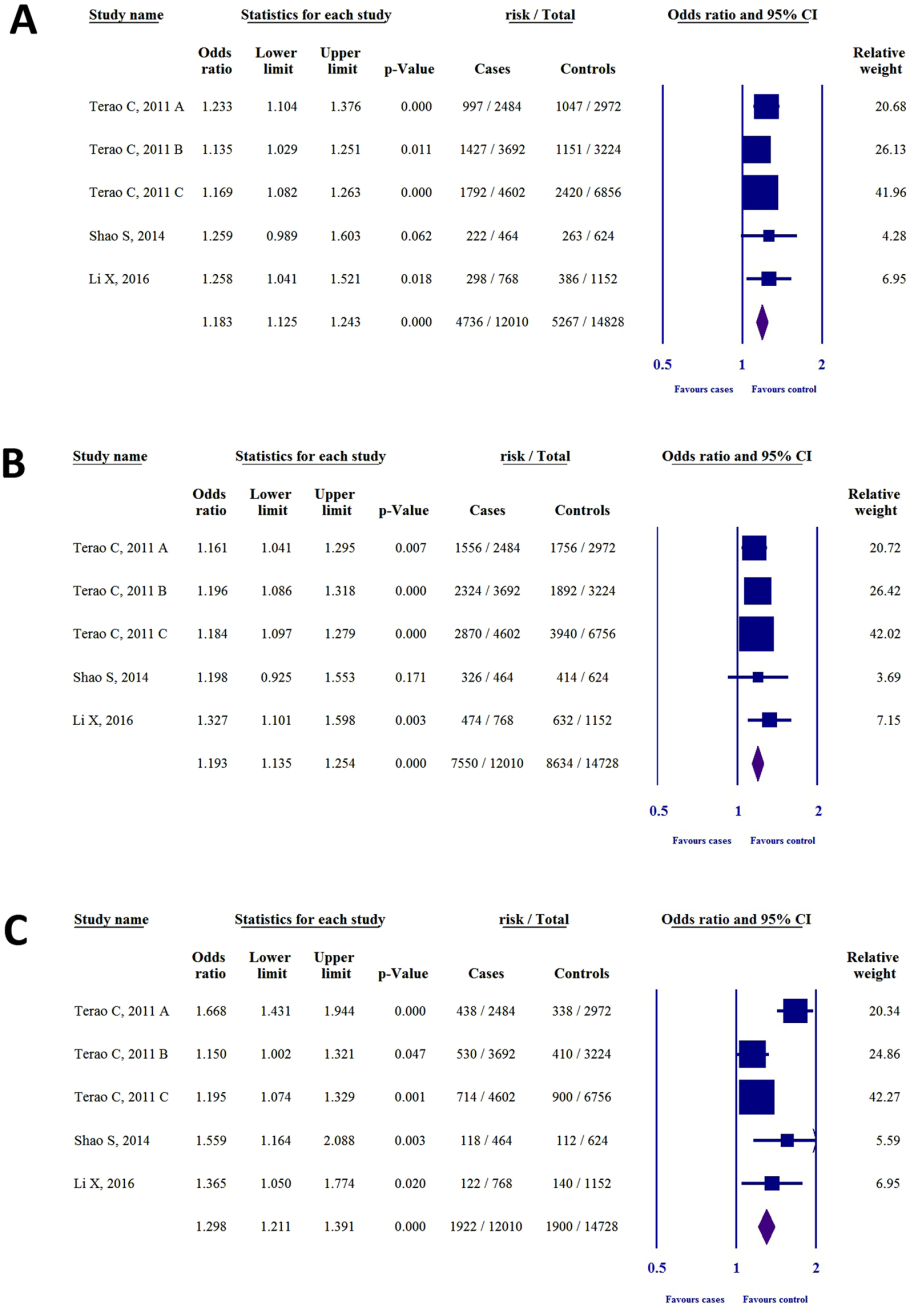
The association of SNP rs760426 (A > G) with RA risk in different genetic models. (**A**) Allelic model (A vs. G). (**B**) Dominant model (AG + AA vs. GG). (**C**) Recessive model (AA vs. AG + GG).

Four studies investigated the association between SNP rs760426 and RA susceptibility^18,20–22^. Most of the publications doubled the individual number; thus, to normalize the data, we also calculated with duplicated values, as was conducted with rs2075876 SNP (see Supplementary Table S1). Again, GWAS by *Terao et al*.^18^ served as three independent case-control studies (denoted with A, B, C). With the exception of *Feng et al*.^21^, all studies described the genotype distribution for AA, GA, GG. We therefore calculated ORs for all the genetic models that were not published in the original articles (Table 2). Furthermore, we excluded the OR, 95% CI and p-value of *Feng et al*.^21^ from the statistical analysis due to the asymmetry of the OR. Results for each genetic model are shown in Fig. 3. For the allelic model (G vs. A), Fig. 3A, the ORs were 1.23 (95% CI 1.10–1.37, P < 0.001), 1.13 (1.02–1.25, P = 0.011), 1.16 (1.08–1.26, P < 0.001), 1.25 (0.98–1.60, P = 0.062), 1.25 (1.04–1.52, P = 0.018). For the dominant model (GG + GA vs. AA, Fig. 3B), the ORs were 1.16 (1.04–1.29, P = 0.007), 1.19 (1.08–1.31, P < 0.001), 1.18 (1.09–1.27, P < 0.001**)**, 1.19 (0.92–1.55, P = 0.171), and OR 1.32 (1.10–1.59, P = 0.003). For the recessive model (GG vs. GA + AA, Fig. 3C) the ORs were 1.66 (95%CI 1.43–1.94, P < 0.001), 1.15 (1.00–1.32, P = 0.047), 1.19 (1.07–1.32, P = 0.001), 1.55 (1.16–2.08, P = 0.003), and 1.36 (1.05–1.77, P = 0.020). For the codominant heterozygous model (GA vs. AA, see Supplementary Fig. S2) the ORs were 1.03, (95% CI 0.92–1.16, P = 0.582), 1.17 (1.06–1.30, P = 0.002), 1.15 (1.06–1.25, P = 0.001), 1.04 (0.79–1.38, P = 0.741), and 1.26 (1.03–1.54, P = 0.020). For the codominant homozygous model (GG vs. AA, see Supplementary Fig. S2), the ORs were 1.69 (1.43–2.00, P < 0.001), 1.25 (1.08–1.46, P = 0.003), 1.29 (1.15–1.44, P < 0.001), 1.60 (1.15–2.24, P = 0.006), and 1.54 (1.16–2.04, P = 0.003).

Results of heterogeneity analysis for each genetic model are shown in Supplementary Table S2. For the allelic model P_h_ = 0.737, I^2^ = 0%, for the dominant model P_h_ = 0.822, I^2^ = 0%, for the recessive model: P_h_ = 0.001, I^2^ = 76.7%, for the codominant heterozygous model: P_h_ = 0.323, I^2^ = 14.2%, and for the codominant homozygous model P_h_ = 0.038, I^2^ = 60.5%. Moderate heterogeneity was found in recessive and codominant homozygous models.

Only four out of 26 ORs were statistically insignificant, and the ORs showed that rs760426 (A > G) SNP is associated an elevated risk. These results therefore suggest a link between *AIRE* SNP rs760426 (A > G) and RA susceptibility.

### Sensitivity analysis

**Figure 4:**
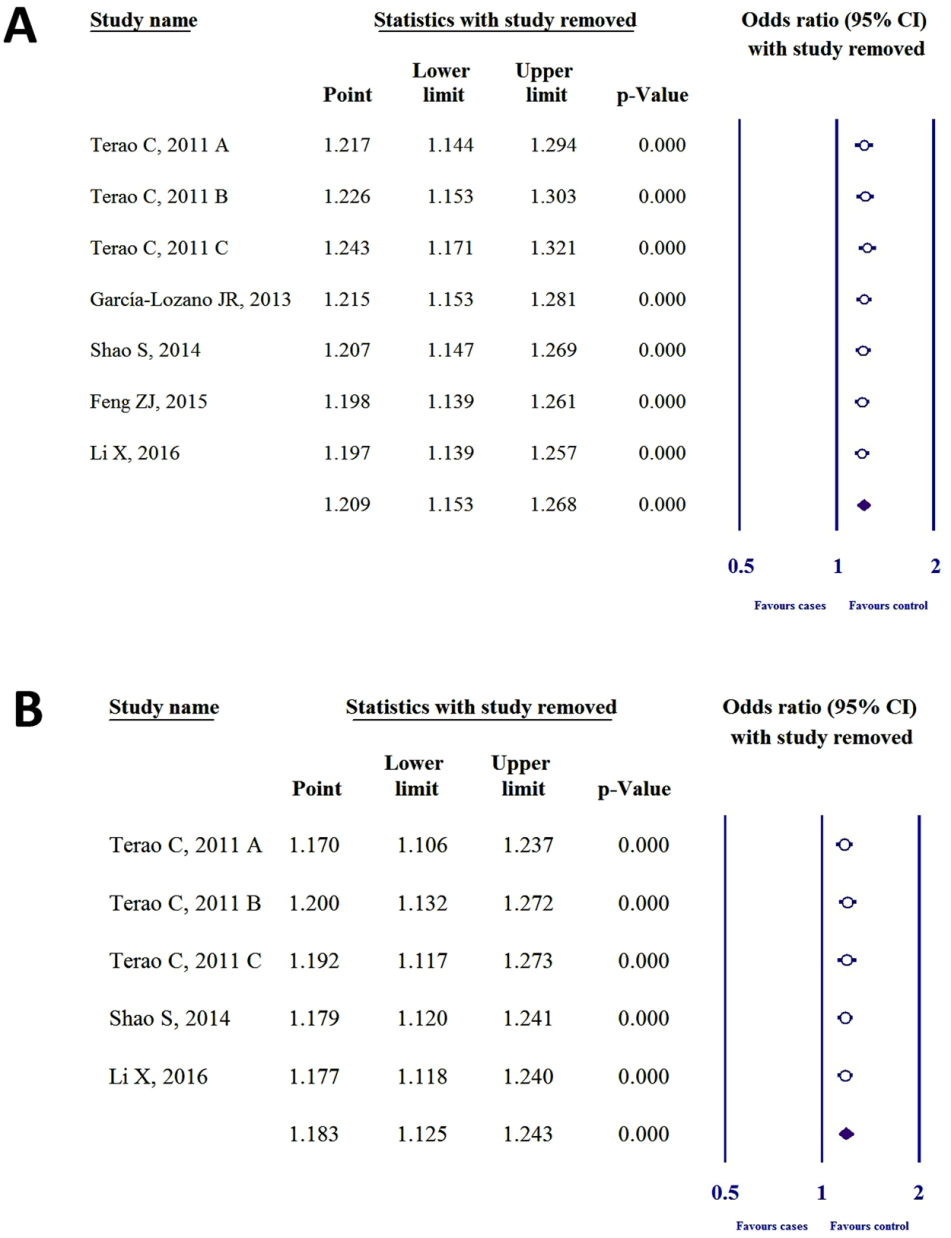
Sensitivity analysis for the allelic models of (**A**) SNP rs2075876 (G > A) and (**B**) rs760426 (A > G).

To detect the influence of each case-control study on the whole meta-analysis, we performed sensitivity analysis by omitting one individual study. Heterogeneity was not found in SNP rs2075876 or rs760426 by investigating allelic (Fig. 4), dominant, recessive, codominant heterozygous, and codominant homozygous genetic models (see Supplementary Figs S3 and S4).

### Publication bias

**Figure 5:**
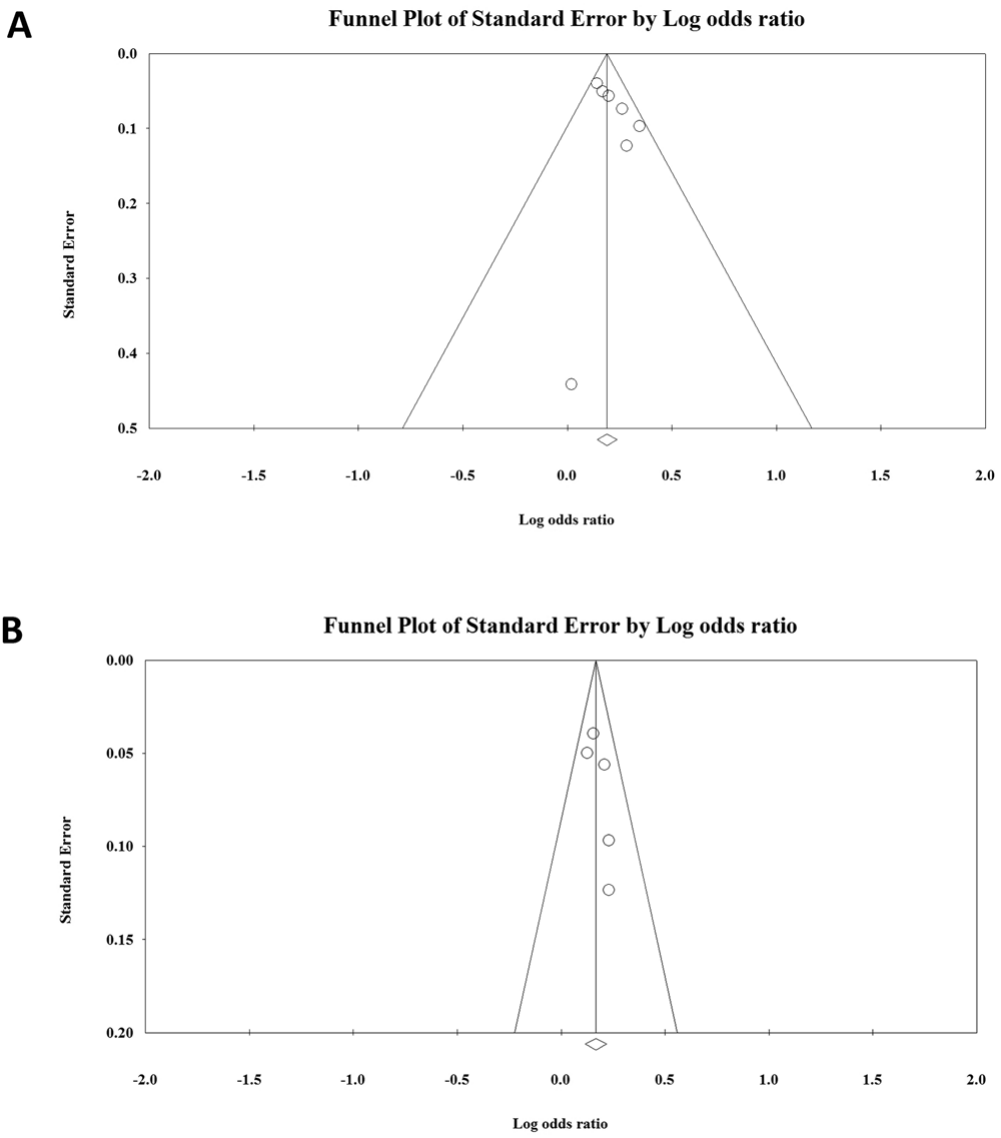
Funnel plots of allelic genetic models of (**A**) SNP rs2075876 (G > A) and (**B**) rs760426 (A > G).

Bias analysis was performed by generating funnel plots for each polymorphism of the allelic (Fig. 5), dominant, recessive, codominant heterozygous, and codominant homozygous genetic models (see Supplementary Figs S5 and S6). After analysis, all funnel plots were perfectly symmetric, and no publication bias was detected for SNP rs2075876 or rs760426.

### Trial sequential analysis

**Figure 6:**
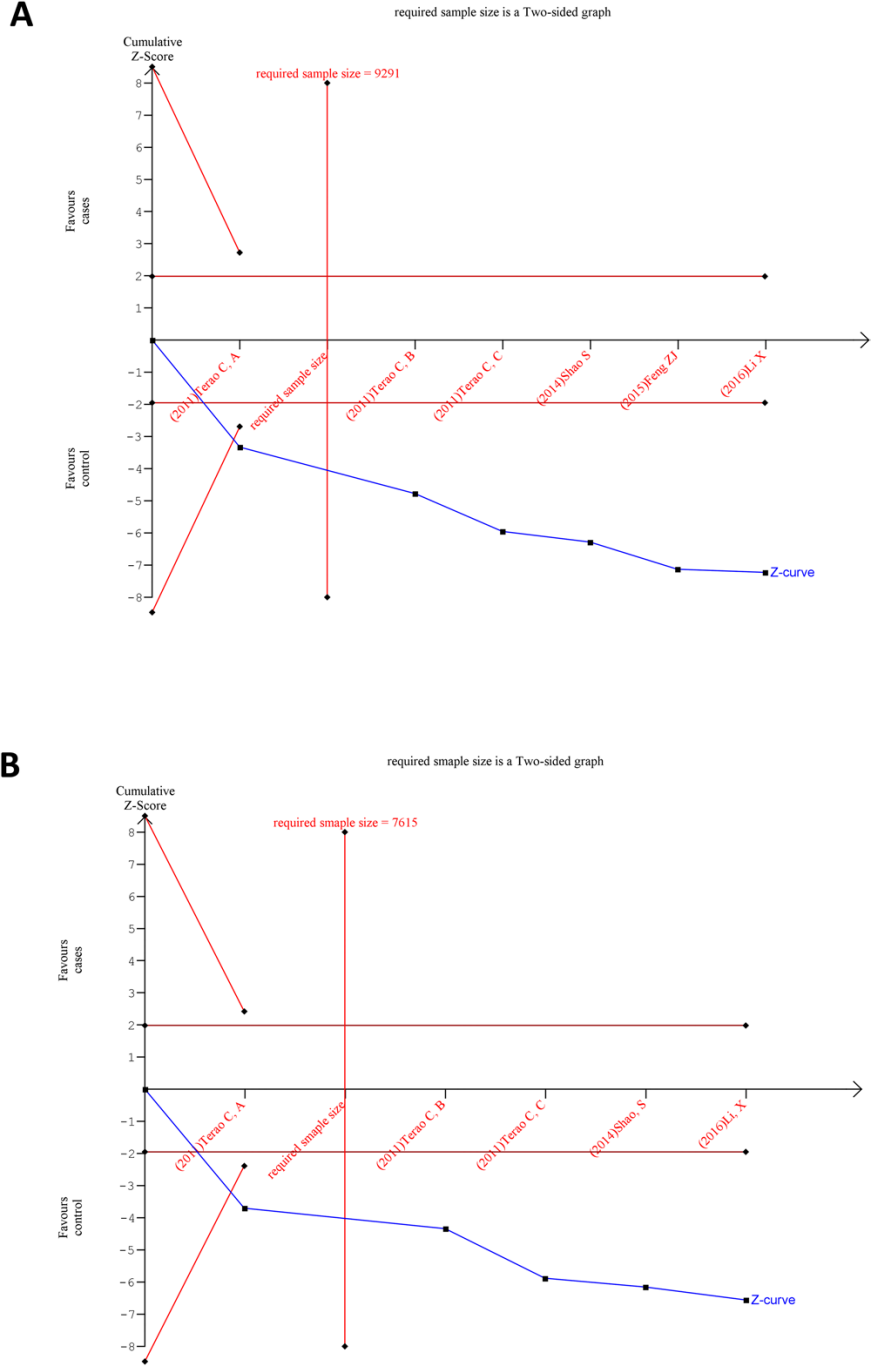
Trial sequential analysis for allelic genetic models of (**A**) SNP rs2075876 (G > A) and (**B**) rs760426 (A > G).

We performed a TSA for the allelic models (Fig. 6) of SNPs rs2075876 (G > A) and rs760426 (A > G). Results of allelic models for both polymorphisms showed that the blue line of cumulative z-curve crossed the TSA monitoring boundary and the cumulative sample size was reached. Therefore, we observed robust evidence in the association between SNPs rs2075876 (G > A) and rs760426 (A > G) and RA risk. These results suggest that no further studies are necessary to confirm the association.

## Discussion

RA is a multifactorial disorder where genetic and environmental events equally contribute to disease commencement^1,25^. The latest GWAS meta-analysis discovered and screened 42 novel RA risk SNPs at a genome level from 98 candidate biological RA risk genes^6^. The detected risk genes, including *AIRE*, are mainly in the category of primary immunodeficiency (PID), HIV, and immune dysregulation. With the exception of AIRE, none of the other associated proteins have been directly related to central tolerance^6^.

Self-tolerance involving negative selection, the machinery of which is directed by AIRE, is a central immuno-physiological process required to create a normal adaptive immune system. We believe that polymorphisms in this indispensable gene lower the protein expression of *AIRE*, decrease the presentation of self-antigens, reduce negative selection, and contribute to the escape and survival of autoimmune T-cells. Reaching the periphery, matured, autoimmune T-cells are a source of autoantibodies and serve as a medium for numerous immune disorders, including RA. In support of this belief, Lovewell *et al*.^13^ have concluded, through a gene reporter assay, that specific haplotypes (*AIRE*−655G *AIRE*-230T) can dramatically reduce *AIRE* transcription. However, with *in vitro* and *in vivo* experiments, Kont *et al*.^26^ have demonstrated that the presentation of PTAs from mTECs is quantitatively affected by these reductions in *AIRE* expression.

An *in silico* investigation by Terao *et al*.^18^, which analysed the expression profile of 210 lymphoblastoid cells in the Gene Expression Omnibus (GEO) database, has demonstrated a statistically significant (p < 0.001) correlation between the rs2075876 risk allele (A) and decreased *AIRE* transcription^27^. No association was found in GEO between rs760426 (G) and *AIRE* expression. Additionally, García-Lozano *et al*.^19^ found statistically significant decreases in the expression levels of rs878081 C allele by analysing GEO database. This SNP is located in the Exon 5 region of *AIRE*; however, rs2075876 (G > A) is located in Intron 5 and rs760426 (A > G) in Intron 12^7^. The latter SNPs may affect the transcription of *AIRE* by modifying alternative splicing or intron-mediated enhancement^28^. The reduction in transcription, in turn, provides lower amounts of PTAs ectopically on the major histocompatibility complex/human leukocyte antigen of mTECs, which thereby contributes to the failure of negative selection in the thymus and increases the survival of autoimmune T-cells. In individuals who carry these SNPs, this sequence increases RA susceptibility. By analysing allelic, dominant, recessive, codominant heterozygous, and codominant homozygous models, we demonstrated that the SNPs rs2075876 (G > A) and rs760426 (A > G) occur more frequently in RA patients than in controls.

There are some limitations in our meta-analysis. We cannot extrapolate the findings of rs2075876 (G > A) and rs760426 (A > G) to Caucasians due to the limited study number. Based on García-Lozano *et al*.^19^, the results are not statistically significant; however, rs878081 C allele seemed to occur more frequently in RA patients. Furthermore, considering GWAS of Terao *et al*.^18^, the association of *AIRE* with RA among Caucasians was not supported. The number of the included studies also limited our meta-analysis; however, Terao *et al*.^18^ provided three case-control studies in one publication, which elevated the number of the included epidemiological studies. In the future, further European case-control, GWAS, and stratified subgroup analyses (age, smoking) are needed in order to better elucidate the association between RA and *AIRE* polymorphism.

To our knowledge, this is the first time that the association between SNP rs2075876 (G > A), rs760426 (A > G), and RA susceptibility was statistically estimated in one meta-analysis. We used multiple haplotype investigations for each polymorphism, sensitivity analyses, and TSA to confirm the robustness of association. In conclusion, our meta-analysis clearly confirmed with each genetic model that the presence of SNPs rs2075876 (G > A) and rs760426 (A > G) is significantly associated with an increased risk for RA.

## Methods

### Search strategy

We searched for related literature in the PubMed, Embase, Cochrane Library, and Web of Science databases in accordance with the recommendations of the Preferred Reporting Items for Systematic Reviews and Meta-Analyses (PRISMA) statement^29^. On 16th May 2017 we completed the search. Keywords (“autoimmune regulator”; “AIRE”; “polymorphism”; “rheumatoid arthritis”) were thoroughly used by two independent investigators. All studies were published from April 2011 to June 2016.

### Inclusion and Exclusion criteria

In order for studies to be included, publications had to demonstrate that (1) the study focused on the association between SNPs or haplotypes within the *AIRE* gene and RA susceptibility, (2) the study was case-control-designed, (3) all RA patients met the American College of Rheumatology classification and diagnostic criteria, and (4) detailed genotype data and feasible ORs, 95% CIs, and p-values were available. Publications were excluded if (1) a previous study was duplicated or (2) the given polymorphism was not found in at least four studies. Review articles were also excluded. Inclusion and exclusion criteria were independently screened by two investigators.

### Statistical analysis

HWE was calculated by the chi-squared test for each study in the control groups. Pooled ORs and 95% CIs were calculated to examine the strength of the association between rs2075876 and rs760426 polymorphisms and RA. We used the random effect model by DerSimonian and Laird^30^ because of the different ethnicities of those included. Heterogeneity between trials was tested with two methods. First, we employed the Cochrane’s Q homogeneity test, which exceeds the upper-tail critical value of chi-square on k–1 degrees of freedom, with a p-value of less than 0.10 considered suggestive of significant heterogeneity. Second, we used the inconsistency (I^2^) index. I^2^ is the proportion of total variation contributed by between-study variability. I^2^ values of 25, 50 and 75% correspond to low, moderate, and high degrees of heterogeneity, respectively, based on Cochran’s handbook^31^. Sensitivity analyses were performed to identify the influence of each study on the pooled ORs and 95% CIs. Publication bias was examined by visual inspection of funnel plots where the standard error was plotted against the log odds ratio. Meta-analytic calculations were performed with Comprehensive MetaAnalysis software Version 3 (Biostat, Inc., Englewood, NJ, USA).

### Trial sequential analysis (TSA)

Meta-analyses may be biased in type I errors owing to an increased risk of random error when sparse data are analysed, combined with reduplicative testing on accumulating data. To avoid this problem and to increase the robustness of conclusions, we used trial sequential analysis (TSA)^32–34^. TSA combines an estimation of the required sample size with an adjusted threshold for statistical significance. The relationship between the cumulative z-curve and the trial sequential monitoring boundary shows the expressiveness of the meta-analysis. If the cumulative z-curve crosses the trial sequential monitoring boundary, and the cumulative sample size of the meta-analysis reaches the required sample size, firm evidence can be observed. When the cumulative z-curve crosses the boundaries, but the sample size does not reach the required information size, a sufficient level of evidence for the anticipated intervention effect may have been reached and no further trials are needed. If the z-curve does not cross any of the boundaries and the required sample size has not been reached, evidence to reach a conclusion is insufficient^35^. For calculation of the information size, we used a heterogeneity adjusted assumption with 10% of relative risk reduction, 5% of overall Type-I-Error, and 10% of Type-II-Error for the case of both gene alleles. The adjusted CIs for rs2075876 and rs760426 are 1.13–1.31 and 1.11–1.26, respectively. For calculations we used the Trial Sequential Analysis software tool from Copenhagen Trial Unit, Center for Clinical Intervention Research, Denmark (version 0.9 beta, www.ctu.dk/tsa).

All data generated or analysed during this study are included in this published article and its Supplementary information file.

## Electronic supplementary material

Dataset 1
