# Supplementary material for: Association between AIRE gene polymorphism and rheumatoid arthritis: a systematic review and meta-analysis of case-control studies
Source: Sci Rep. 2017 Oct 26;7:14096. doi: 10.1038/s41598-017-14375-z (PMC5658331; doi:10.1038/s41598-017-14375-z)
Supplement: Supplementary file 1 — Dataset 1 [file 41598_2017_14375_MOESM1_ESM.doc]

**Association between AIRE gene polymorphism and rheumatoid arthritis: a systematic review and meta-analysis of case-control studies**

Bálint Bérczi^1^, Gellért Gerencsér^1^, Nelli Farkas^2^, Péter Hegyi^3,4,5^, Gábor Veres^6^, Judit Bajor^7^, László Czopf^8^, Hussain Alizadeh^9^, Zoltán Rakonczay^10^, Éva Vigh^11^, Bálint Erőss^4^, Kata Szemes^7^, Zoltán Gyöngyi^1^

^1^Department of Public Health Medicine, Medical School, University of Pécs, Pécs, Hungary. ^2^Institute of Bioanalysis, Medical School, University of Pécs, Pécs, Hungary. ^3^MTA-SZTE Translational Gastroenterology Research Group, Szeged, Hungary. ^4^Institute for Translational Medicine, University of Pécs, Pécs, Hungary. ^5^Department of Translational Medicine, First Department of Medicine, University of Pécs, ^6^1st Department of Pediatrics, Semmelweis University, Budapest Hungary. ^7^Department of Gastroenterology, First Department of Medicine, University of Pécs, Pécs, Hungary. ^8^Division of Cardiology and Angiology, First Department of Medicine, University of Pécs, Pécs, Hungary. ^9^Department of Haematology, First Department of Medicine, University of Pécs, Pécs, Hungary. ^10^Department of Pathophysiology, University of Szeged, Szeged, Hungary. ^11^Department of Radiology, Medical School, University of Pécs, Pécs, Hungary. Correspondence and requests for materials should be addressed to Z.G. (zoltan.gyongyi@aok.pte.hu)

**Supplementary Table S1. Genetic distributions of the included study populations and Hardy-Weinberg Equilibrium (HWE) of the control populations for the rs2075876 (G>A) and rs760426 (A>G)**

| **polymorphism** | **study** | | **group** | **sample size** | **minor allele** | **major allele** | **genotype distribution** | | | **HWE** |
| --- | --- | --- | --- | --- | --- | --- | --- | --- | --- | --- |
|  |  |  |  |  | **A** | **G** | **GG** | **AG** | **AA** |  |
| ***SNP rs2075876***  ***(G>A)*** | Terao C, 2011 | A | cases | 2470 | 956 | 1514 | 960 | 1108 | 402 |  |
|  |  |  | controls | 2972 | 1014 | 1958 | 1278 | 1360 | 334 | 0.49 |
|  |  | B | cases | 3672 | 1373 | 2299 | 1412 | 1774 | 486 |  |
|  |  |  | controls | 3146 | 1055 | 2091 | 1420 | 1342 | 384 | 0.08 |
|  |  | C | cases | 4592 | 1721 | 2871 | 1810 | 2122 | 660 |  |
|  |  |  | controls | 6732 | 2302 | 4430 | 2924 | 3012 | 796 | 0.73 |
|  | García-Lozano JR, 2013 | | cases | 942 | NA | | | | |  |
|  |  |  | controls | 930 |  |  |  |  |  | NA |
|  | Shao S, 2014 | | cases | 464 | 226 | 238 | 122 | 232 | 110 |  |
|  |  |  | controls | 626 | 261 | 365 | 210 | 310 | 106 | 0.74 |
|  | Feng ZJ, 2015 | | cases | 1382 | 686 | 696 | 346 | 700 | 336 |  |
|  |  |  | controls | 1600 | 690 | 910 | 546 | 728 | 326 | 0.59 |
|  | Li X, 2016 | | cases | 768 | 307 | 461 | 278 | 366 | 124 |  |
|  |  |  | controls | 1152 | 369 | 783 | 526 | 514 | 112 | 0.55 |
|  |  | |  |  | **G** | **A** | **AA** | **GA** | **GG** |  |
| ***SNP***  ***rs760426***  ***(A>G)*** | Terao C, 2011 | A | cases | 2484 | 997 | 1487 | 928 | 1118 | 438 |  |
|  |  |  | controls | 2972 | 1047 | 1925 | 1216 | 1418 | 338 | 0.07 |
|  |  | B | cases | 3692 | 1427 | 2265 | 1368 | 1794 | 530 |  |
|  |  |  | controls | 3224 | 1151 | 2073 | 1332 | 1482 | 410 | 0.96 |
|  |  | C | cases | 4602 | 1792 | 2810 | 1732 | 2156 | 714 |  |
|  |  |  | controls | 6756 | 2420 | 4336 | 2816 | 3040 | 900 | 0.21 |
|  | Shao S, 2014 | | cases | 464 | 222 | 242 | 138 | 208 | 118 |  |
|  |  |  | controls | 624 | 263 | 361 | 210 | 302 | 112 | 0.89 |
|  | Feng ZJ, 2015 | | cases | 1382 | NA | | | | |  |
|  |  |  | controls | 1600 |  |  |  |  |  | 1.0 |
|  | Li X, 2016 | | cases | 768 | 298 | 470 | 294 | 352 | 122 |  |
|  |  |  | controls | 1152 | 386 | 766 | 520 | 492 | 140 | 0.31 |

**Supplementary Figure S1. The association of SNP rs2075876 (G>A) with RA risk in different genetic models. A) Codominant heterozygous model (GA vs. AA). B) Codominant homozygous model (GG vs. AA)**


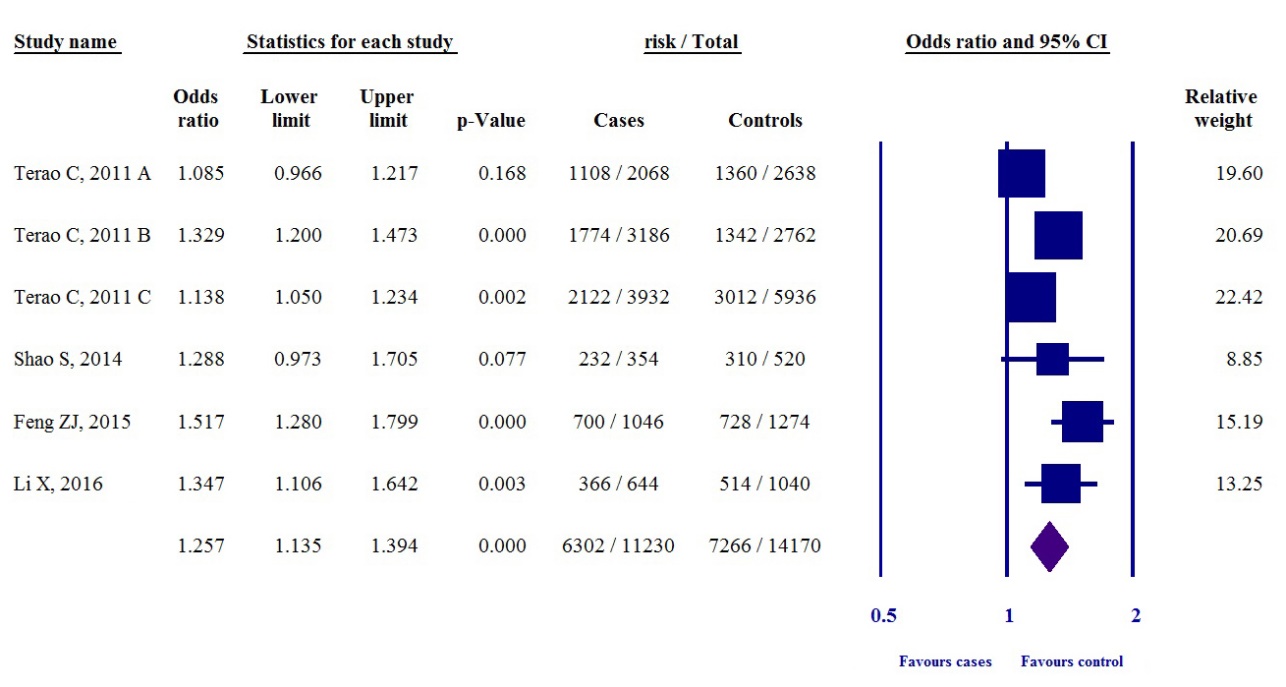


**A**

**B**


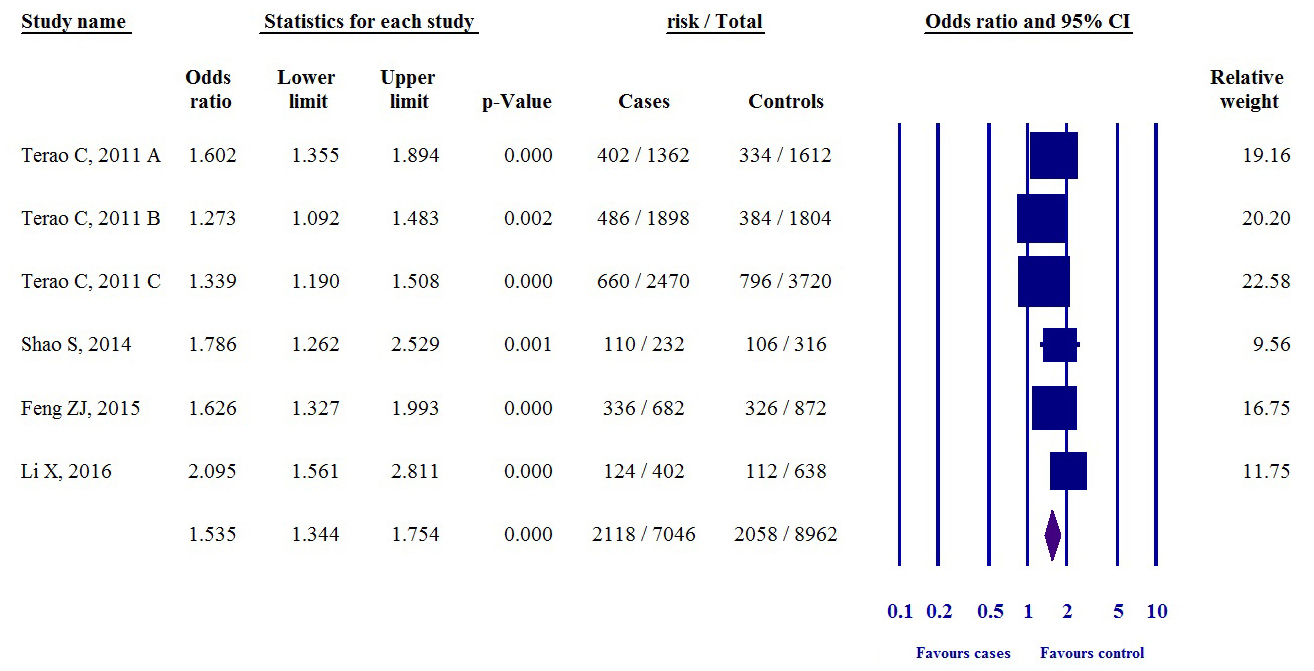


**Supplementary Figure S2. The association of SNP rs760426 (A>G) with RA risk in different genetic models. A) Codominant heterozygous model (AG vs. GG). B) Codominant homozygous model (AA vs. GG). CI=confidence interval.**


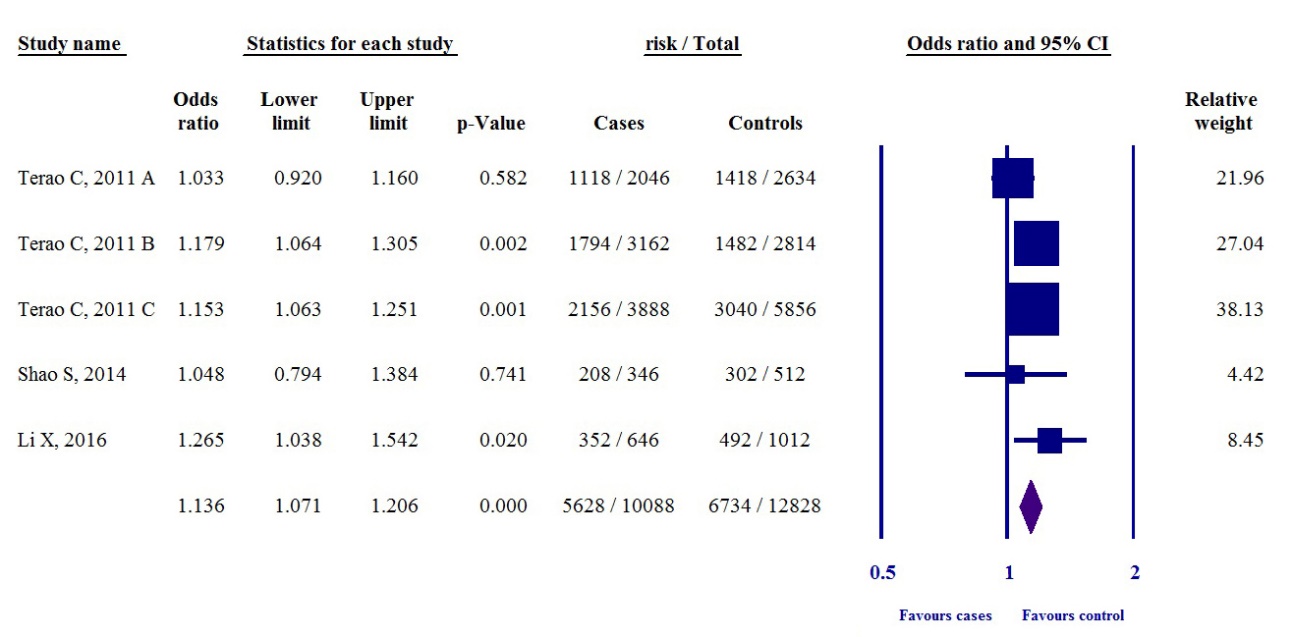

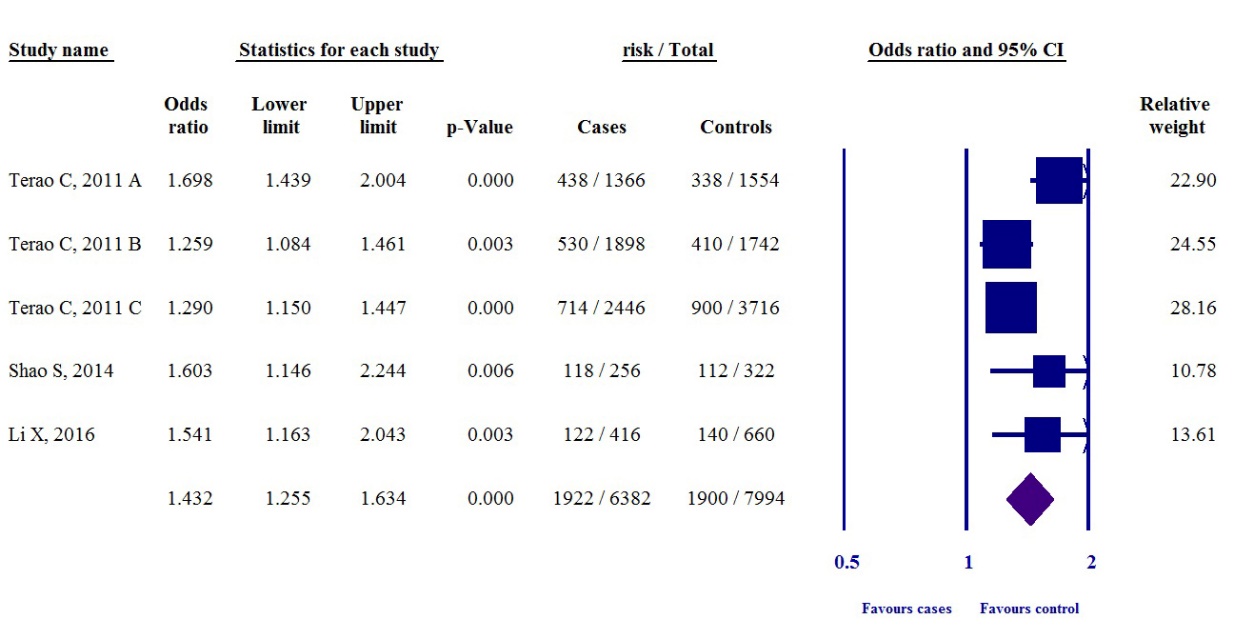


**A**

**B**

**Supplementary Table S2. Pooled ORs, 95%CI, P-values and the results of heterogeneity analysis (Q-value, P_(heterogeneity)_, I^2^) for each genetic models of SNP rs2075876 (G>A) and rs760426 (A>G).**

|  | **Genetic model** | **OR** | **95%CI** | **P** | **Q-value** | **P _(heterogeneity)_** | **I^2^ (%)** |
| --- | --- | --- | --- | --- | --- | --- | --- |
| ***SNP***  ***rs2075876***  ***(G>A)*** | Allelic | 1.20 | 1.15-1.26 | < 0.001 | 5.85 | 0.439 | 0 |
|  | Dominant | 1.31 | 1.20-1.44 | < 0.001 | 14.82 | 0.011 | 66.2 |
|  | Recessive | 1.35 | 1.18-1.53 | < 0.001 | 16.65 | 0.005 | 69.9 |
|  | Codominant heterozygous | 1.25 | 1.13-1.39 | < 0.001 | 16.98 | 0.004 | 70.5 |
|  | Codominant homozygous | 1.53 | 1.34-1.75 | < 0.001 | 14.47 | 0.012 | 65.4 |
| ***SNP***  ***rs760426***  ***(A>G)*** | Allelic | 1.18 | 1.12-1.24 | < 0.001 | 1.98 | 0.737 | 0 |
|  | Dominant | 1.19 | 1.13-1.25 | < 0.001 | 1.52 | 0.822 | 0 |
|  | Recessive | 1.29 | 1.21-1.39 | < 0.001 | 17.20 | 0.001 | 76.7 |
|  | Codominant heterozygous | 1.13 | 1.07-1.20 | < 0.001 | 4.66 | 0.323 | 14.2 |
|  | Codominant homozygous | 1.43 | 1.25-1.63 | < 0.001 | 10.13 | 0.038 | 60.5 |

**Supplementary Figure S3. Sensitivity analysis for A) dominant, B) recessive, C) codominant heterozygous and D) homozygous genetic models of SNP rs2075876 (G>A).**


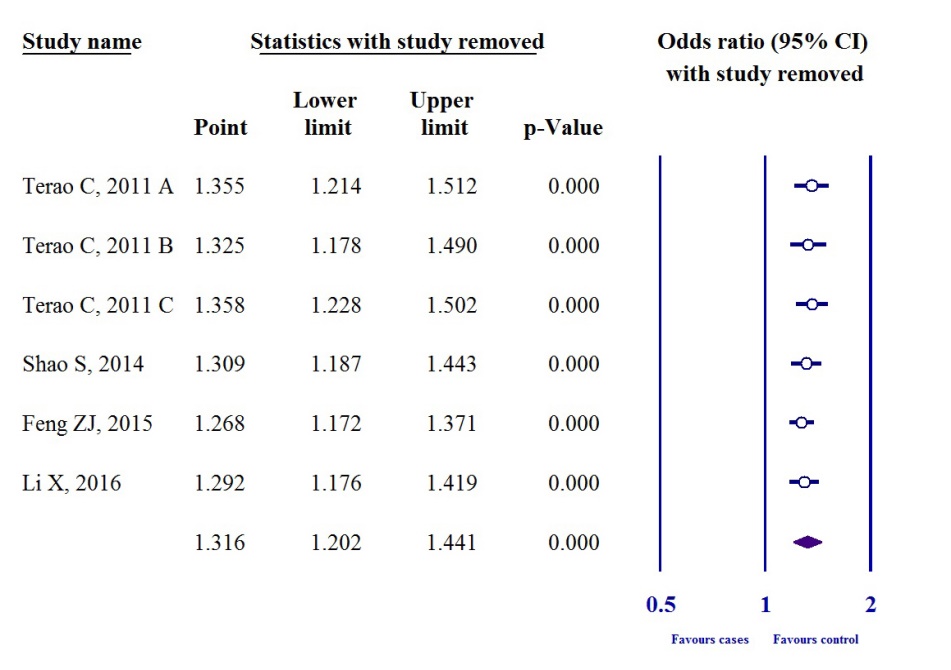


**A**


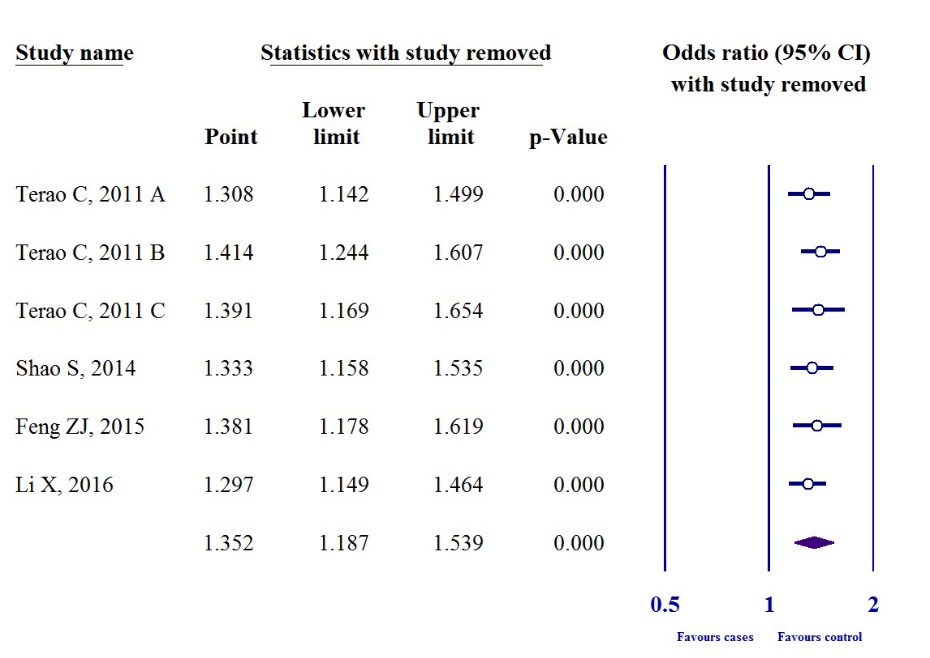


**B**


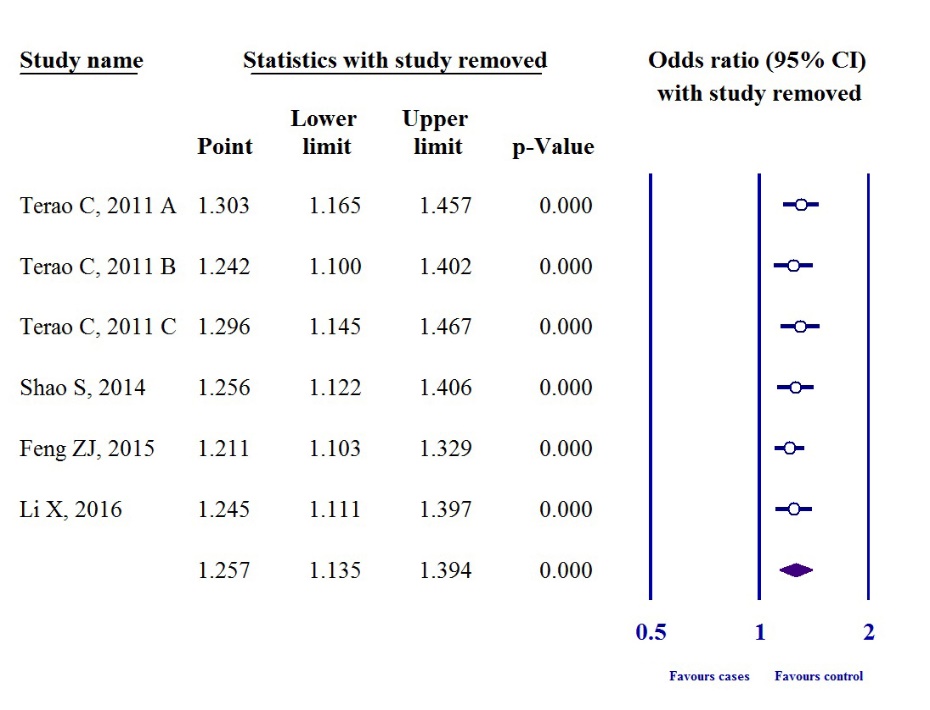


**C**


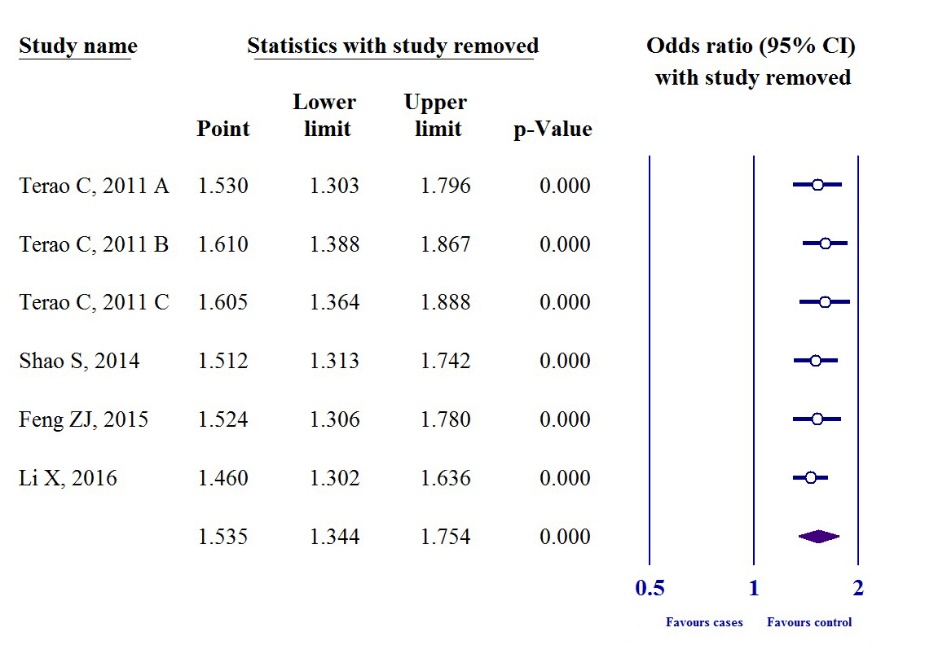


**D**

**Supplementary Figure S4. Sensitivity analysis for A) dominant, B) recessive, C) codominant heterozygous and D) homozygous genetic models of SNP rs760426 (A>G).**

**
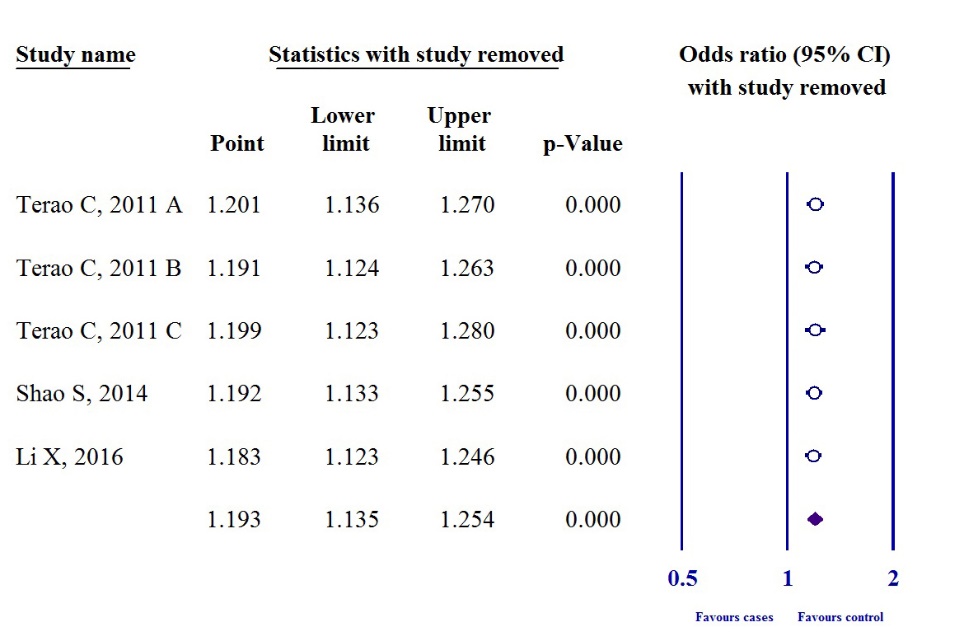
**

**A**


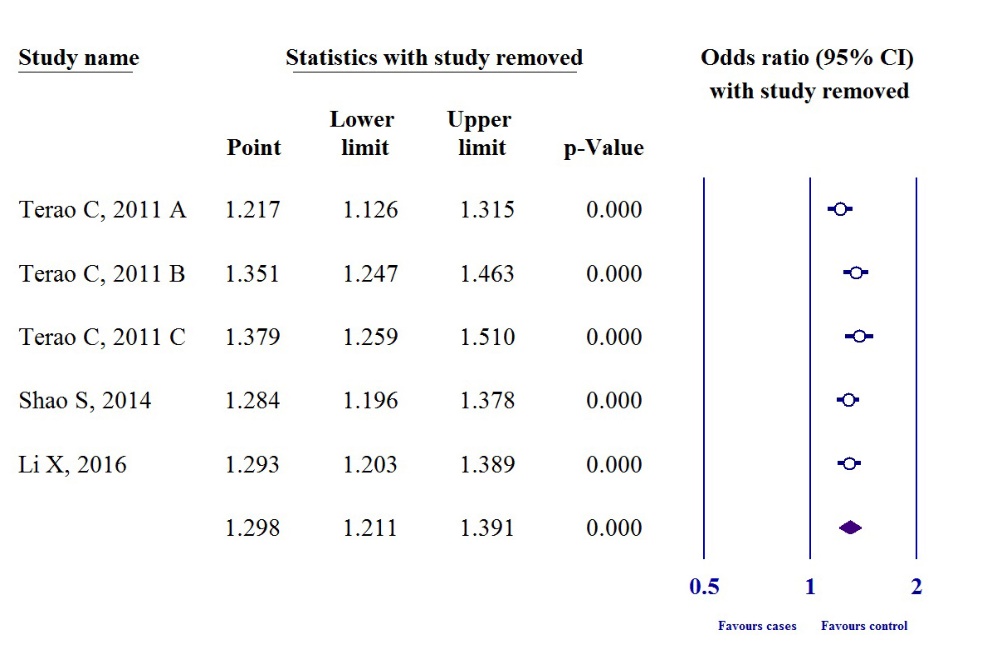

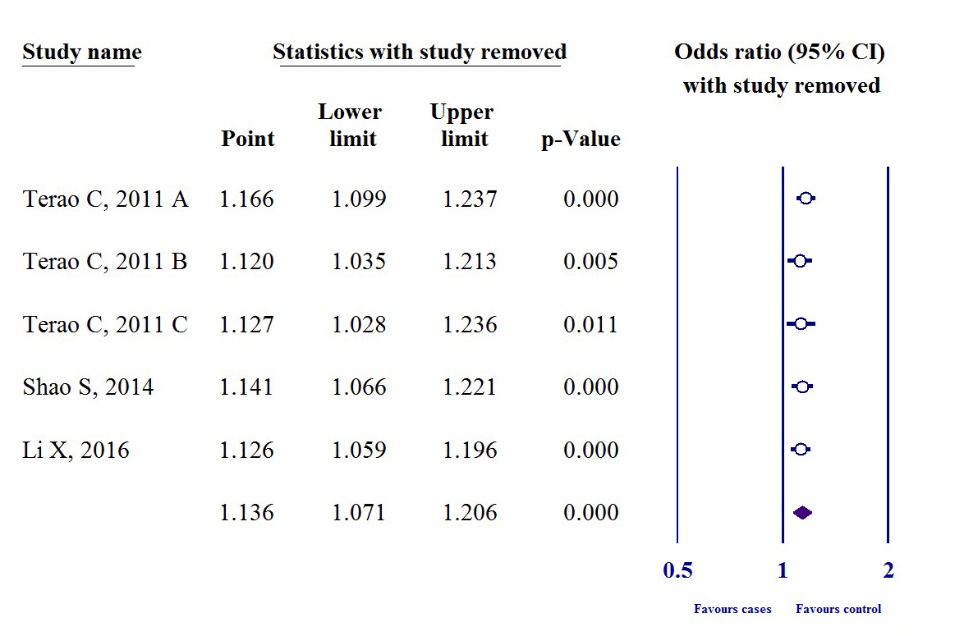


**B**

**C**


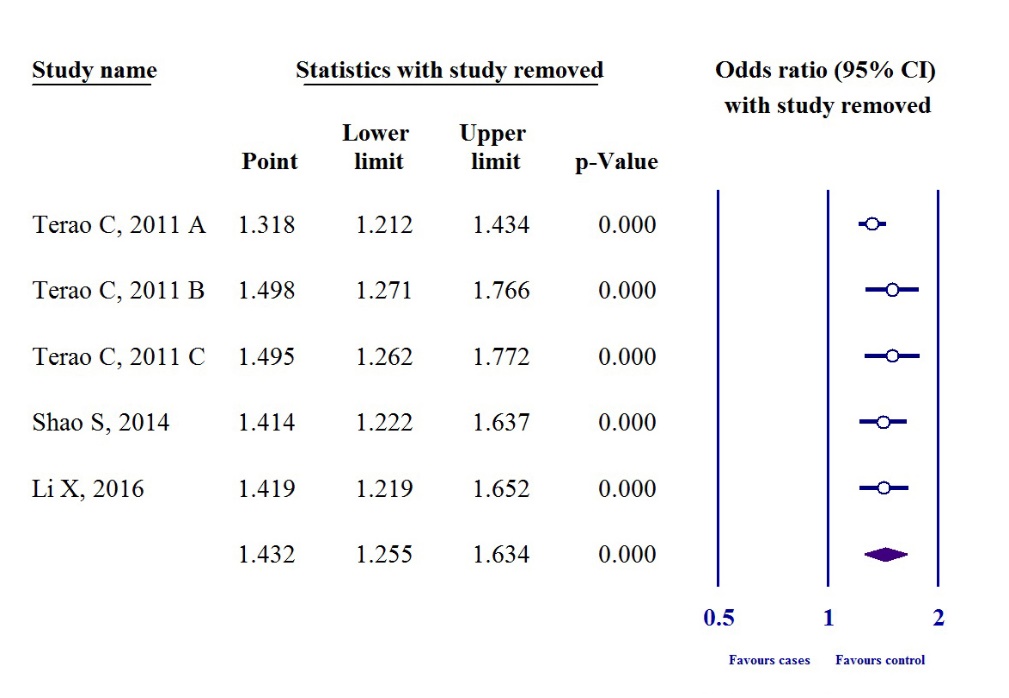


**D**

**Supplementary Figure S5. Funnel plots of A) dominant, B) recessive, C) codominant heterozygous and D) homozygous genetic models of SNP rs2075876 (G>A).**

**
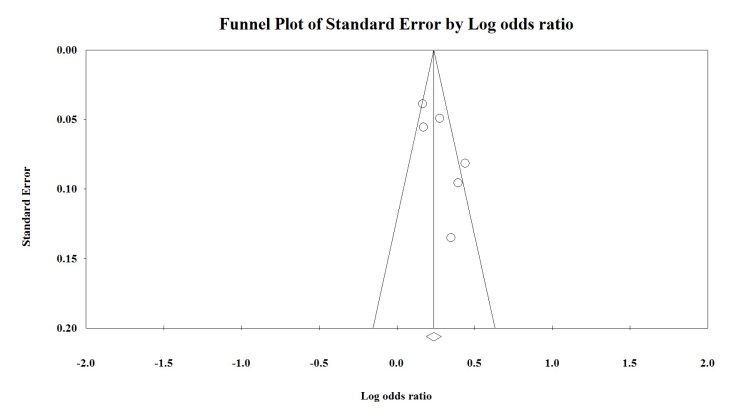

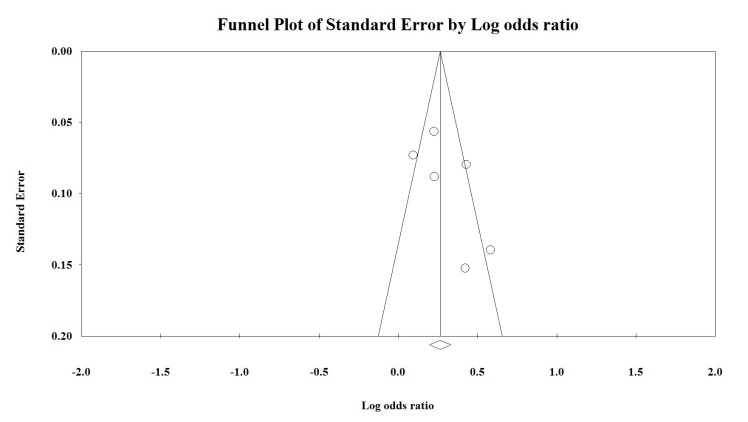

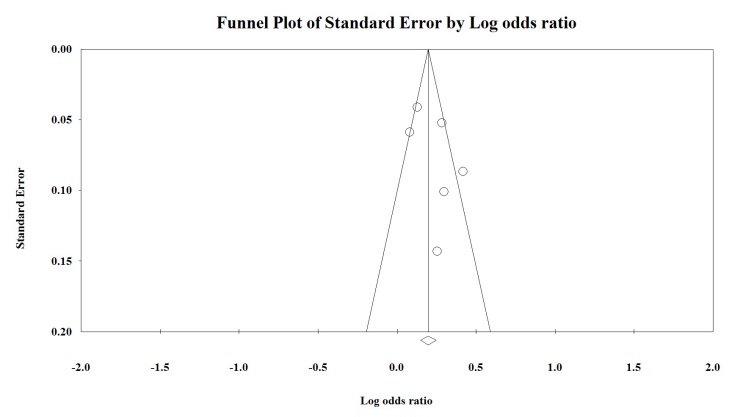

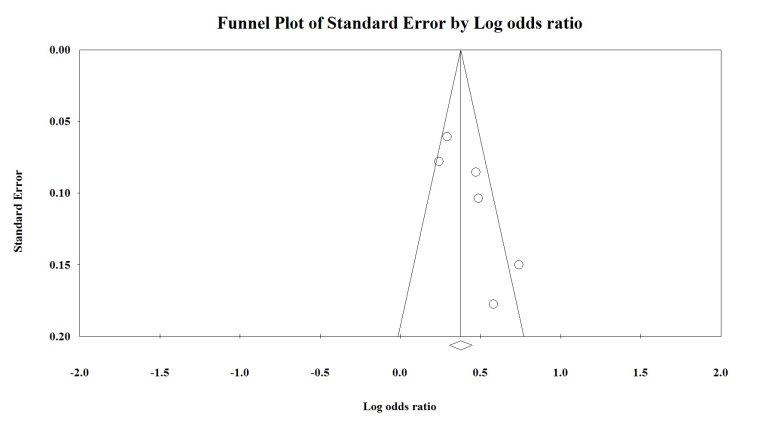
**

**A**

**B**

**C**

**D**

**Supplementary Figure S6. Funnel plots of A) dominant, B) recessive, C) codominant heterozygous and D) homozygous genetic models of SNP rs760426 (A>G).**

**
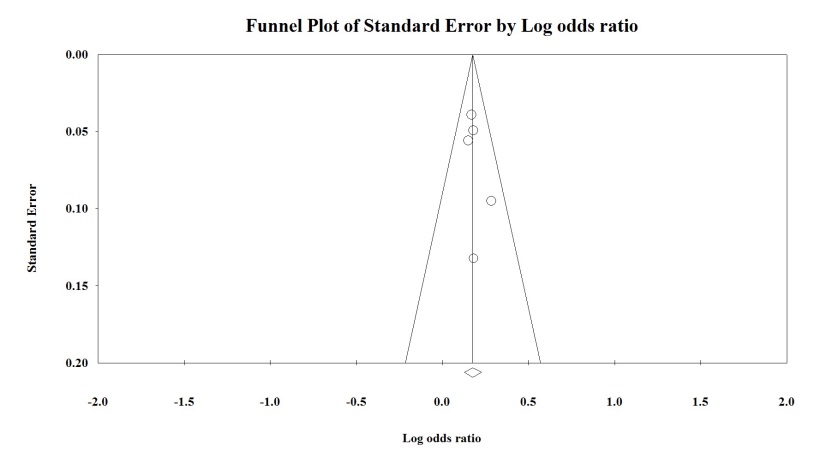

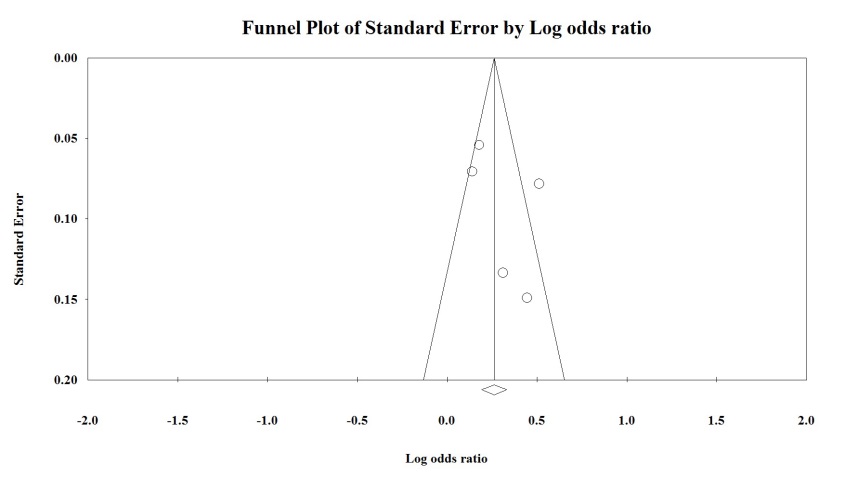

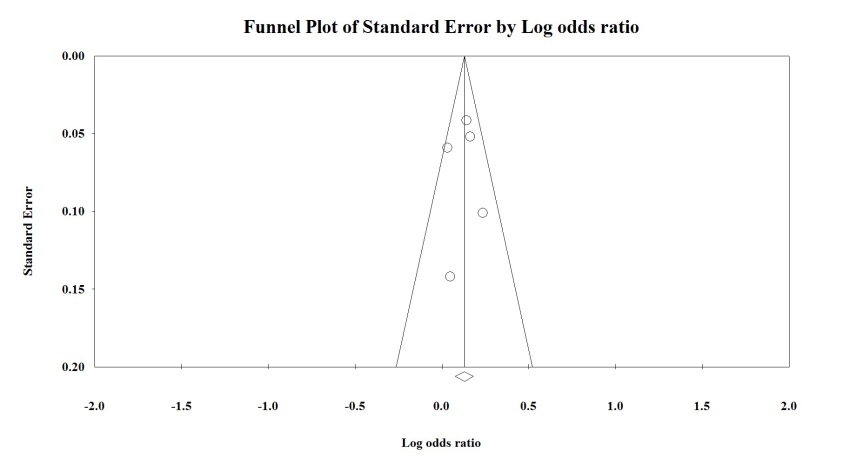

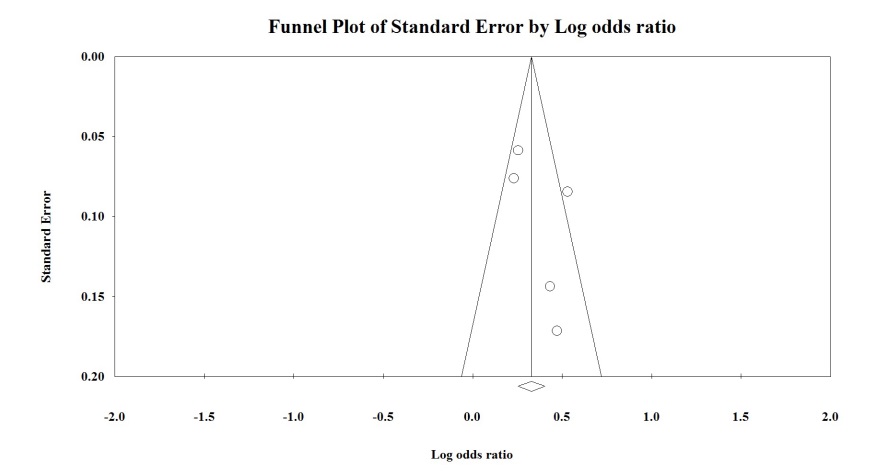
**

**A**

**B**

**C**

**D**
